# Antibiofilm and Antioxidant Activity of Propolis and Bud Poplar Resins versus* Pseudomonas aeruginosa*

**DOI:** 10.1155/2017/5163575

**Published:** 2017-01-03

**Authors:** Stefania De Marco, Miranda Piccioni, Rita Pagiotti, Donatella Pietrella

**Affiliations:** Biochemical Sciences and Health Section, Department of Pharmaceutical Sciences, University of Perugia, Via del Giochetto, 06122 Perugia, Italy

## Abstract

*Pseudomonas aeruginosa* is a common biofilm-forming bacterial pathogen implicated in lung, skin, and systemic infections. Biofilms are majorly associated with chronic lung infection, which is the most severe complication in cystic fibrosis patients characterized by drug-resistant biofilms in the bronchial mucus with zones, where reactive oxygen species concentration is increased mainly due to neutrophil activity. Aim of this work is to verify the anti-*Pseudomonas* property of propolis or bud poplar resins extracts. The antimicrobial activity of propolis and bud poplar resins extracts was determined by MIC and biofilm quantification. Moreover, we tested the antioxidant activity by DPPH and neutrophil oxidative burst assays. In the end, both propolis and bud poplar resins extracts were able to inhibit* P. aeruginosa* biofilm formation and to influence both swimming and swarming motility. Moreover, the extracts could inhibit proinflammatory cytokine production by human PBMC and showed both direct and indirect antioxidant activity. This work is the first to demonstrate that propolis and bud poplar resins extracts can influence biofilm formation of* P. aeruginosa* contrasting the inflammation and the oxidation state typical of chronic infection suggesting that propolis or bud poplar resins can be used along with antibiotic as adjuvant in the therapy against* P. aeruginosa* infections related to biofilm.

## 1. Introduction

Propolis (bee glue) is the generic name for the resinous substance collected by honey bees (*Apis mellifera* L.) from various plant sources (substances exuded from wounds in plants: lipophilic materials on leaves and leaf buds, gums, resins, latices, etc.); it is used to seal holes in the honeycombs and smooth out the internal walls. Propolis is also used to protect the entrance against intruders; moreover, it contains antimicrobial agents active against a variety of pathogens.

In the temperate zone all over the world, the main source of bee glue is the resinous exudate of the buds of poplar trees, mainly the black poplar* Populus nigra*. European propolis contains phenolics: flavonoid aglycones (flavones and flavanones), phenolic acids, and their esters [1, 2].

Propolis from tropical regions has a different chemical composition; for example, Brazilian bee glue is harvested from the leaf resin of* Baccharis dracunculifolia* and it is composed of prenylated derivatives of* p*-coumaric acid and of acetophenone. Diterpenes, lignans, and flavonoids (different from those in “poplar type” propolis) have also been found [3]. The Cuban propolis from the floral resin of* Clusia rosea* is composed mainly of polyisoprenylated benzophenones [4].

The chemical composition of propolis is qualitatively and quantitatively variable, depending on the geographic and regional plant ecology. Propolis is widely used in traditional medicine and is reported to have a broad spectrum of pharmacological effects: antibacterial, antihepatotoxic, antioxidative, anti-inflammatory, and so forth. It has been demonstrated that flavonones, flavones, phenolic acids, and their esters of European propolis have antimicrobial, anti-inflammatory, and antioxidant activity, whereas the antibacterial, antioxidant, and antitumoral activity of the Brazilian propolis are ascribed to prenylated p-coumaric acids, labdane diterpenes, and flavonoids. The antitumor activity of the European propolis is indeed attributed to the caffeic acid phenethyl ester [4].

Poplar buds are coated with a resin that contains different phenolic compounds as terpenoids, flavonoid aglycones, and their chalcones and phenolic acids and their esters [5]. Bud resins are mixed with bee salivary enzymes and beeswax in the final propolis. Then, propolis and poplar bud exudates have the same ingredients but the composition can be different.

*Pseudomonas aeruginosa* is an opportunistic pathogen with a particular ability to cause disease in the immunocompromised subjects.* P. aeruginosa* is the most common Gram-negative bacterium found in nosocomial infections where water systems have been reported to contribute to bacterial transmission [6]. Moreover,* P. aeruginosa* is a cause of life-threatening infections in cystic fibrosis (CF) patients: the 54.4% of the whole CF patient population is infected with this bacterium, which is found in 80% of the patients by the age of 18 years [7].

In nature, most bacterial genus, among which* Pseudomonas*, is likely to form biofilm attached to biotic and abiotic surfaces as a survival strategy [8]. Biofilms are of considerable medical importance because of their involvement in persistent infections [9]. Sessile bacteria show enhanced resistance to conventional antibiotics and host defenses. Within a biofilm matrix, bacteria are able to resist antibiotics at concentrations up to 1000–1500 times higher than that conventionally used [10].

Many antibiofilm compounds against this bacterium have been identified from diverse natural sources, some of them impairing the bacterial quorum sensing, such as the synthetic derivate of natural furanone [11] or garlic extract [12]. Other antibiofilm natural compounds include five ursine triterpenes and corosolic acid from* Diospyros dendo* and asiatic acid [10–12], ginseng aqueous extract and its constituent zingerone [13, 14], tannins from* Anadenanthera colubrina*,* Commiphora leptophloeos*, and* Myracrodruon urundeuva* [15] and bacterial products such as 3-indolylacetonitrile [16]. In addition, a few plant extract libraries have been used to control* P. aeruginosa* biofilm formation [17–19].

Thus, to understand if the bee salivary enzyme can influence the biological effects of resin components the aim of this study was to assess the antimicrobial activity versus* P. aeruginosa*, and the antioxidant and anti-inflammatory effects of propolis and bud poplar resins ethanol extracts. Moreover, the phytochemical analysis of the samples was performed; the cytotoxic concentration (CC_50_) of both extracts was evaluated on HeLa, BEAS-2B, and A549 epithelial cell lines and human monocytes. Finally, the anti-inflammatory activity of propolis and bud poplar resins assessing cytokine production was analyzed. Both extracts have shown antibiofilm activity. Bud poplar resins extract showed a better antioxidant and anti-inflammatory capacity at noncytotoxic concentration than propolis extract.

## 2. Materials and Methods

Absolute ethanol 99.8% was purchased from Sigma-Aldrich. Ultrahigh purified water used in this study was prepared in a Purelab Ultra water purification system (ELGA, UK). Pinocembrin (cod.P5239) and chrysin (cod.95082) were purchased from Sigma-Aldrich. Galangin (cod.114S) was purchased from Extrasynthese. Caffeic acid phenethyl ester (CAPE) (Sigma, cod. C8221), was purchased from Sigma-Aldrich.

### 2.1. Propolis: Origin and Preparation

Propolis has been gently provided by ABOCA S.p.A. (Italy). In the present study, propolis and bud poplar resins extracts in 85% ethanol were analyzed. Italian propolis was smashed with a pestle and dissolved in ethanol 85% at a ratio of 1/5 w/v. Mixture was then extracted for 8 h by shacking at 30–40°C. Poplar buds were grounded by an electronic blender before extraction. Samples were then centrifuged; supernatants were recovered, concentrated, and lyophilized. Samples were maintained at 4°C. A stock solution of each propolis has been prepared in ethanol at a concentration of 100 mg/mL and maintained at 4°C.

### 2.2. Analysis of Total Flavonoids Expressed as Galangin

The finely ground sample (0.20 g of poplar buds or propolis freeze-dried extract) was extracted in ethanol 85% (50 mL) during 30 minutes in an ultrasound bath at a temperature of 35 ± 5°C. The extract was then centrifuged at 4000 rpm for 5 minutes, the supernatant was transferred into a 100 mL volumetric flask, and the residue was treated at the same conditions. The combined extracts were brought to a final volume of 100 mL. The absorption of the sample, diluted 1 : 50 v/v, was recorded at 353 nm by means of quartz cuvettes, using ethanol 85% as reference solution. As *A*_1%,1 cm_ of galangin at 353 nm is 600.67, the % value of the total flavonoids was calculated.

### 2.3. Analysis of Pinocembrin, Galangin, and Chrysin

The samples, extracted as described above, were then analyzed by means of an UHPLC 1290 (Agilent Technologies INC., Santa Clara, CA) system equipped with a vacuum degasser, a binary pump, a Peltier thermostated autosampler at 10°C, and a Peltier thermostated column compartment and the effluent was analyzed by a DAD detector at 220 nm. The column used was a Phenyl-Hexyl (Poroshell, Agilent, 3.0 mm × 100 mm, 2.7 *μ*m) maintained at 40°C. The elution was performed with H_2_O/H_3_PO_4_ 0.2% (solvent A) and Acetonitrile (solvent B). The gradient program used was 0–12.5 min 70% A, 30% B flow rate 0.5 mL/min; 12.5–16 min 70% A, 30% B flow rate 0.36 mL/min; 16–22 min 70% A, 30% B flow rate 0.36 mL/min; 22–26 min 20% A, 80% B flow rate 0.5 mL/min; 26–28 min 70% A, 30% B flow rate (0.5 mL/min). Chrysin, galangin, and pinocembrin were quantified using pure substances as external standards. The concentration was calculated by means of a calibration curve in the range of 0.1–0.02 mg/mL.

### 2.4. Analysis of Caffeic Acid Phenethyl Ester (CAPE)

The samples extracted as described above were analyzed by means of a HPLC 1260 (Agilent Technologies INC., Santa Clara, CA) system equipped with a vacuum degasser, a binary pump, a Peltier thermostated autosampler at 10°C, and a Peltier thermostated column compartment and the effluent was analyzed by a DAD detector at 220 nm. The column used was a C18 (Prodigy ODS3, Phenomenex, 250 × 4.6 mm 5 *μ* equipped with a security guard C18, 4 × 3 mm 5 *μ*) maintained at 60°C. The elution was performed with H_2_O/H_3_PO_4_ 0.2% (solvent A) and Acetonitrile (solvent B). The gradient program used was 0–43 min 70% A, 30% B; 43–45 min 20% A, 80% B; 45–50 min 20% A, 80% B; 50–55 min 70% A, 30% B. The flow rate was 1 mL/min. Caffeic acid phenethyl ester was quantified using pure substance as external standard. The concentration was calculated by means of a calibration curve in the range of 0.05–0.01 mg/mL.

### 2.5. Microorganisms

*P. aeruginosa* (P1242) expressing the luciferase gene and luciferin substrate under the control of a constitutive P1 integron promoter was generously gifted by Choi and Schweizer [20].* P. aeruginosa* (P1242) and* P. aeruginosa* PAO1 (ATCC 15692) were used. All experiments were conducted in Muller Hinton Broth (MHB) at 37°C. Bacteria were initially streaked from −80°C glycerol stock onto a Muller Hinton Agar (MHA) plate and a fresh single colony was inoculated into MHB (15 mL) and cultured at 37°C. Overnight cultures were then inoculated into medium at a dilution of 1 : 100. Cell growths were determined by measuring optical densities at 600 nm using a spectrophotometer (Infinite M200 pro, TECAN).

### 2.6. Minimal Inhibitory Concentration (MIC) Assay

The Minimal Inhibitory Concentration (MIC) was determined by microbroth dilution method according to the Clinical and Laboratory Standards Institute/National Committee for Clinical Laboratory Standards (CLSI/NCCLS) Approved Standard M100-S21, 2007 [21]. Gentamicin solution (2 mg/mL) was prepared by dissolving the agent in endotoxin free water. Solutions of propolis and bud poplar resins extracts (100 mg/mL) were prepared in ethanol. Briefly, to determine the MIC of propolis and bud poplar resins extracts or Gentamicin, MHB was used. Extracts were diluted in MHB; the dilutions, ranging from 0.9 to 2000 *μ*g/mL, were prepared in U bottom 96-well plates. The inoculum size of bacteria was about 0.5 McFarland. The plates were incubated at 37°C for 24 h. The MIC of each extracts was defined as the lowest concentration that inhibited visible growth of the organism.

### 2.7. Minimal Bactericidal Concentration (MBC) Assay

The Minimal Bactericidal Concentration (MBC) was determined as the lowest concentration of Gentamicin or propolis and bud poplar resins extracts at which no microbial growth was observed. For the MBC determination, Muller Hinton Agar plates were seeded with 10 *μ*L of cell suspensions taken from the wells of the plates of MIC assay, where cell growth was not observed. These plates were incubated at 37°C for 24 h and colony forming units (CFU) growth was evaluated.

### 2.8. Growth Curve Inhibition

The antimicrobial activity of propolis and bud poplar resins extracts against* P. aeruginosa* was investigated at three different concentrations (100, 50, and 10 *μ*g/mL). Test was carried out in 96-well culture plates. 200 *μ*L of microbial suspensions in MHB (10^5^ cells/mL) was incubated at 37°C in a microplate reader (Infinite M200 pro, TECAN). Every two hours of incubation luminescence of each well was analyzed. Each analysis was performed in triplicate. Results are presented as the mean of relative luminescence units (RLU).

### 2.9. Propolis Effect on Biofilm Formation

The in vitro static biofilm assay was performed using a 96-well microtiter plate as previously described with some modification [22]. To grow biofilms, overnight cultures of* P. aeruginosa* were diluted 1 : 100 into fifteen mL of Tryptic Soy Broth (TSB) supplemented with 2% sucrose, in presence or in absence of different extracts tested at the concentrations indicated below. Cultures were incubated at 37°C for 24 h under static conditions. After incubation, the biofilm developed in each well was washed twice with 200 *μ*L of distilled water and then dried for 45 min. In each well, 100 *μ*L of 0.4% crystal violet was added for 30–45 min. After this procedure, the wells were washed four times with distilled water and immediately discolored with 200 *μ*L of 95% ethanol. After 45 minutes, 100 *μ*L of discolored solution was transferred to a well of a new plate and the crystal violet was measured at 570 nm in a microplate reader (Infinite M200 pro, TECAN). The amount of biofilm formed was measured comparing the absorbance values of the compounds-treated wells versus untreated control wells. Biofilm formation bioassays were performed in triplicates in at least three individual experiments for each concentration.

### 2.10. Swimming Motility

Swimming motility was performed as previously described [14]. Media plates containing 1.0% tryptone, 0.5% NaCl, and 0.3% agarose were point inoculated with sterile toothpick from overnight culture of* P. aeruginosa* P1242 grown with and without supplementation of propolis and bud poplar resins extracts. After incubation at 30°C for 24 h, swimming motility was determined by measuring the radius of circular expansion of bacterial migration from the point of inoculation.

### 2.11. Swarming Motility

Swarming motility was performed as previously described [14]. Nutrient agar (8.0 g/L) supplemented with glucose (5.0 g/L) was prepared and plates were point inoculated with sterile toothpick from overnight culture of* P. aeruginosa* P1242 grown with and without supplementation of propolis and bud poplar resins extracts. After incubation at 37°C for 24 h, swarming motility was determined by measuring circular turbid zones.

### 2.12. Twitching Motility

Twitching motility was performed as previously described [14]. Media plates containing agar layer of Luria broth (1.0% agar) were prepared and stabbed with toothpick up to bottom of the Petri dish from overnight culture of* P. aeruginosa* P1242 grown with and without supplementation of propolis and bud poplar resins extracts. After incubation at 37°C for 48 h, a hazy zone of growth at the interface between the agar and polystyrene surface was observed.

### 2.13. Cell Lines

A549 (human lung adenocarcinoma epithelial cell line, ATCC CCL-185), BEAS-2B cells (derived from normal bronchial epithelium obtained from autopsy of noncancerous individuals and infected with a replication-defective SV40/adenovirus 12 hybrid, ATCC CRL-9609), HeLa cells (human cervix adenocarcinoma epithelial cell line, ATCC CCL-2), HuDe (human dermis fibroblast cell line, Istituto Zooprofilattico Sperimentale della Lombardia e dell'Emilia Romagna, BS PRC 41), and NCTC2544 cells (human skin keratinocytes, Istituto Nazionale per la Ricerca sul Cancro HL97002) were used in this study. The culture medium consisted of RPMI 1640 with 2 mM glutamine, 10% FBS (fetal bovine serum), and 100 U (units) penicillin and 100 *μ*g streptomycin/mL, referred as cRPMI. Confluent cultures were split using 0.25% trypsin/EDTA. Monolayers were incubated at room temperature for 5–10 min until cell detachment. Fresh medium was added to disperse cells and suspensions were then centrifuged and adjusted at the desired concentration in culture medium.

### 2.14. Peripheral Human Mononuclear (PBMC) and Polymorphonuclear (PMN) Cells Isolation

Heparinized venous blood was obtained from buffy coat gently provided by Blood Bank of the Ospedale della Misericordia of Perugia. All donors have been informed and they signed the consensus form (MO-SIT_06) approved by Ethics Committee CEAS (Comitato Etico Aziende Sanitarie) (Rev. 3 Ottobre 2014) in which they authorize the use of their sample for research studies. Heparinized venous blood was diluted with RPMI 1640 (Gibco-BRL). Human peripheral blood mononuclear cells (PBMC) were separated by density gradient centrifugation over Ficoll-Hypaque Plus (Pharmacia Biotech), recovered, washed twice, and suspended in RPMI 1640 supplemented with 10% FBS, 100 U penicillin/mL, and 100 *μ*g streptomycin/mL. The pellet containing PMN and erythrocytes was treated with hypotonic saline to lyse the erythrocytes. Granulocytes were collected by centrifugation, washed twice in RPMI 1640, counted, and adjusted to the desired concentration.

### 2.15. Cytotoxicity Assay

The cytotoxicity was tested by the determination of the cell ATP level by ViaLight® Plus Kit (Lonza). The method is based upon the bioluminescent measure of ATP that is present in all metabolically active cells. The bioluminescent method utilizes the luciferase, an enzyme which catalyzes the formation of light from ATP and luciferin. The emitted light intensity is linearly related to the ATP concentration and it is measured using a luminometer. To perform cytotoxicity tests, cells were recovered, counted and adjusted to the concentration of 2 × 10^5^/mL, seeded in a flat bottom 96-well culture plate, and incubated until monolayer formation. The examinations were carried out for propolis and the control (cells not treated). Various 1 : 2 dilutions of the above-mentioned propolis and bud poplar resins extracts were prepared in cRPMI in order to achieve the following final concentrations in the wells: 2000, 1000, 500, 250, 125, 62.5, 31, 16, 8, 4, 2, and 1  *μ*g/mL. Each concentration was tested in triplicate. After adding extracts onto cell monolayers, plates were incubated for 24 h at 37°C. After incubation, plates were left to cool at room temperature for 10 minutes and then the Cell Lysis Reagent was added to each well to extract ATP from cells. Next, the AMR Plus (ATP Monitoring Reagent Plus) was added and after 2 more minutes the luminescence was read using a microplate luminometer (TECAN). Results are expressed as 50% cytotoxic concentration (CC_50_), the concentration required to reduce the cell viability by 50% compared to the untreated controls.

### 2.16. Cytokine Determination

PBMC were stimulated with lipopolysaccharide (LPS 1 *μ*g/mL, Sigma) for 4 h and then propolis extracts were added to the cultures at the indicated concentrations for further 24 h. Dexamethasone was added as positive control at the concentration of 25 *μ*g/mL. Culture supernatants were recovered and stored at −20°C until cytokine determination. TNF-*α* and IL-1*β* were determined using an immune assay ELISA (U-Cytech Biosciences, Utrecht, Netherlands).

### 2.17. DPPH Radical Scavenging Activity

The antioxidant activity of propolis and bud poplar resins extracts was evaluated by using the 2,2-diphenyl-1-picrylhydrazyl (DPPH) free radical scavenging assay as described by Dutra et al. [23] with some modifications. The extracts were diluted in ethanol at 10.0 and 100.0 *μ*g/mL and added to an ethanol solution of DPPH (50.0 *μ*g/mL). After 5, 10, and 30 min of reaction at room temperature in the dark, the absorbance of each solution was read at 517 nm in a spectrophotometer (TECAN). The mixture of ethanol and sample was used as blank. The control solution was prepared by mixing ethanol and DPPH radical solution. Ascorbic acid was used as positive control. The percent inhibition was calculated using the following formula:

(1) DPPH scavenging activity%=100−A  sample−A  blank×100A  control,

where *A* sample equals absorbance of the sample after 30 min of reaction, *A* blank equals absorbance of the blank, and *A* control equals absorbance of the control.

### 2.18. Evaluation of ROS Production by Chemiluminescence Assay

Antioxidant activity was evaluated by chemiluminescence assay according to Fernandes et al. [24] with small modifications. Chemiluminescence measurements were performed in a final volume of 0.25 mL. 50 *μ*L of luminol (0.28 mM) and 50 *μ*L of different concentrations of the propolis and bud poplar resins extracts were added to 100 *μ*L of neutrophil solution (1.25 × 10^6^ cells/mL) and the mixture was incubated for 3 min at 37°C. The cells were then stimulated with 50 *μ*L of 10^−7^ M phorbol-12-myristate-13-acetate (PMA). The chemiluminescence produced by the cells was monitored for 20 min in a luminometer (TECAN), in which the light output was recorded as RLU (relative photons units). Each measure was performed in triplicate.

### 2.19. Statistical Analysis

All experiments were performed in triplicates in at least three different experiments. Data were expressed as mean ± SD of data obtained from different experiments. Differences between propolis-treated biofilm and untreated biofilm were compared using the Student's *t*-test (two-tailed). *P* values of < 0.05 were considered significant.

## 3. Results

### 3.1. Propolis: Marker Analysis

**Figure 1:**
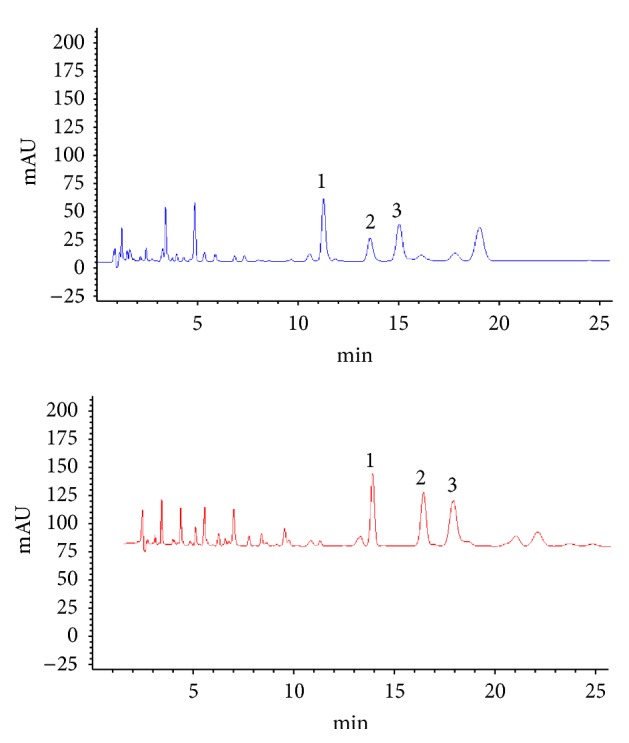
Phytochemical analysis of propolis and bud poplar resins. Chromatogram of poplar bud resins extract (blue line) and propolis extract (red line). Chrysin (1), galangin (2), and pinocembrin (3) were quantified using pure substances as external standards. The concentration was calculated by means of a calibration curve in the range of 0.1–0.02 mg/mL.

Propolis and bud poplar resins extracts underwent phytochemical characterization and some compounds were quantified by means of specific methods illustrated in Section 2. The content in chrysin, galangin, and pinocembrin has been detected by HPLC (Figure 1). The chromatogram shows that galangin concentration is higher in propolis extracts than in bud poplar resins extracts.

Moreover, results in Table 1 showed that propolis has a higher content of total flavonoids, chrysin, and galangin than poplar bud resins, while both extracts showed a similar content of pinocembrin and caffeic acid.

### 3.2. Antimicrobial Activity

The initial determination of the anti-*Pseudomonas* activity of propolis was performed in vitro by standardized CLSI/NCCLS methods [21] and this was done against both strains of* P. aeruginosa* used throughout this study. The MIC of the two different extracts against* P. aeruginosa* genetically modified* P1242* and the wild type strain PAO-1 was 125 *μ*g/mL (Table 2). The determination of the MBC suggests that the effect of propolis and bud poplar resins extracts is bacteriostatic and not bactericidal. No significant differences have been noticed between the two strains of* Pseudomonas* suggesting that the genetic modification did not influence the antimicrobial sensitivity of the strain.

**Figure 2:**
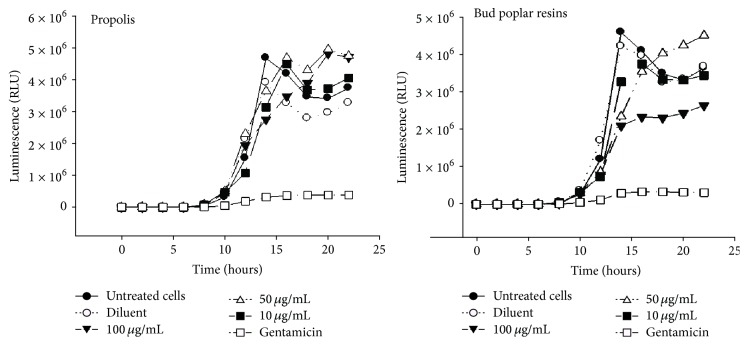
Growth curve of* Pseudomonas aeruginosa* P1242 in presence of propolis and bud poplar resins extracts.* P. aeruginosa* cells were grown in presence or absence of extracts at 10, 50, and 100 *μ*g/mL for 24 h at 37°C. Gentamicin (1.8 *μ*g/mL) was used as positive control. Luminescence of live cells in the culture is expressed as relative luminescence units (RLU).

Kinetics growth of bacteria has been studied by determination of luminescence of the culture that is correlated with alive bacteria. Three different sub-MIC concentrations of propolis (100, 50, and 10 *μ*g/mL) have been tested. Results in Figure 2 show that only the bud poplar resins extracts delayed the bacterial growth at the concentration of 100 *μ*g/mL. Gentamicin, used as positive control, inhibited the growth of bacteria.

**Figure 3:**
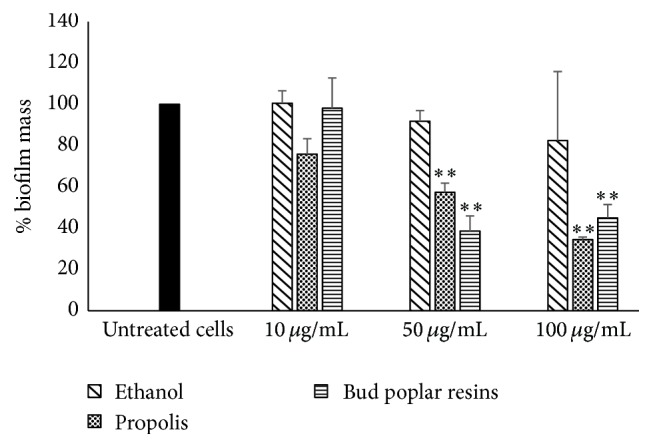
Effect of propolis and bud poplar resins extracts on biofilm formation.* P. aeruginosa* biofilm was developed in presence or absence of different extracts (10, 50, and 100 *μ*g/mL) or diluent (same concentration present in the propolis solutions) for 24 h at 37°C. Biofilm biomass was quantified by crystal violet assay (absorbance 570 nm). ^*∗∗*^*P* < 0.01 (biofilm grown in the presence of propolis or bud poplar resins extracts versus biofilm formed in presence of diluent).

*P. aeruginosa* is an opportunistic pathogen that is capable of colonizing various human tissues and organs and is often resistant to many currently used antibiotics. This resistance is often caused by the ability to form biofilms. The new approaches proposed to combat bacterial infections consider the attenuation of virulence and then the inhibition of biofilm formation. Then, the ability of propolis and bud poplar resins extracts to inhibit the biofilm formation has been detected. Each extract was tested at 100, 50, and 10 *μ*g/mL. Results, reported in Figure 3, show that both propolis and bud poplar resins extracts are able to reduce biofilm formation with respect to biofilm formed in the presence of the diluent at 50 and 100 *μ*g/mL.

**Figure 4:**
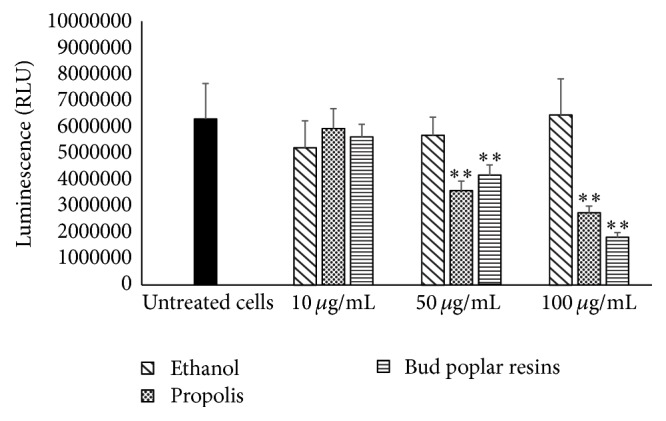
Effect of propolis and bud poplar resins extracts on sessile* Pseudomonas aeruginosa*.* P. aeruginosa* biofilm was developed in presence or absence of different extracts (10, 50, and 100 *μ*g/mL) or diluent (same concentration present in the propolis solutions) for 24 h at 37°C. ^*∗∗*^*P* < 0.01 (sessile bacteria in biofilm formed in the presence of propolis and bud poplar resins extracts versus sessile bacteria in biofilm formed in presence of diluent).

To determine the amount of alive bacteria entrapped into the biofilm, the luminescence of* P. aeruginosa* has been tested. Results in Figure 4 show a reduced number of alive* P. aeruginosa* cells in biofilm grown in the presence of both propolis and bud poplar resins extracts, suggesting that the reduction of biofilm mass is due to a diminished amount of sessile bacteria.

### 3.3. Mechanisms of Biofilm Inhibition

Recently, the role of swimming flagellar motility, twitching motility (mediated by type IV pili, which extend and retract to tug bacterial cells across the surface), and swarming in* P. aeruginosa* biofilm formation has been reviewed [25]. The first step in biofilm formation is the reversible attachment to a surface. To overcome surface repulsion,* P. aeruginosa* utilizes flagellar-mediated swimming motility [25]. After interaction with the surface,* P. aeruginosa* can move using twitching motility or by swarming, which utilizes the flagellum, as well as surfactants, to migrate on a substratum. In order to verify the role of propolis and bud poplar resins extracts in* Pseudomonas* motility we tested swimming, twitching, and swarming activity in presence of both extracts.

**Figure 5:**
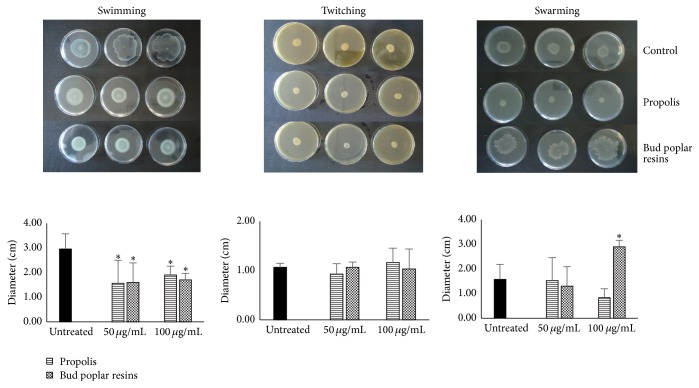
Swimming, twitching, and swarming motility of* Pseudomonas aeruginosa* in presence of propolis or bud poplar resins extracts. Swimming, twitching, and swarming motility were determined by measuring the radius of bacterial colony, hazy or turbid zone. ^*∗*^*P* < 0.05 (colonies grown in the presence of propolis versus colonies formed in presence of diluent).

Both extracts were able to inhibit partially the swimming activity of* P. aeruginosa*. A reduced swimming motility increases the repulsion strength of surface inhibiting the formation of a stable adhesion. To establish irreversible attachment and progress to the formation of a mature biofilm,* P. aeruginosa* represses both twitching and swarming motility subsequent to cell-to-surface contact (Figure 5). Bud poplar resins extract is able to increase swarming motility, inhibiting the progression of biofilm maturation process. No effect on twitching activity by both extracts has been observed.

### 3.4. Cell Viability

Cytotoxicity of the different extracts tested was evaluated by using an ATP-bioluminescence kit (Via Light kit, Cambrex) in three cell lines: human cervix adenocarcinoma epithelial cells (HeLa), human bronchial cells (BEAS-2B), and human lung adenocarcinoma epithelial cells (A549). Moreover, cytotoxicity was evaluated on fresh human PBMC. All in vitro experiments were repeated in triplicate. According to the data presented in Table 3, both extracts have similar effects on viability of the different human cells. However, different cell lines showed a different susceptibility.

### 3.5. Antioxidant Activity

**Figure 6:**
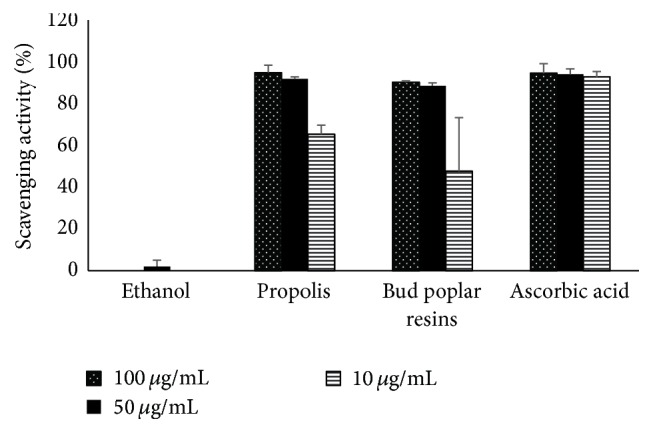
Antioxidant activity of propolis and bud poplar resins extracts. Results are expressed as % of DPPH scavenging activity. Data represent the mean ± SD of 2 or more independent experiments performed in triplicate.

In a series of experiments, we analyzed the antioxidant ability of propolis and bud poplar resins extracts by means of neutralizing the free DPPH radical. Significant differences in the ability to scavenge DPPH by both extracts were observed (Figure 6). The concentration of 10 *μ*g/mL of propolis and bud poplar resins extracts were able to neutralize the 65,4% and 47.6% of DPPH, respectively, but the difference between the two extracts was not significant (*P* > 0.05). Doses of 50 and 100 *μ*g/mL of both extracts showed an activity comparable to that observed for the positive control ascorbic acid.

**Figure 7:**
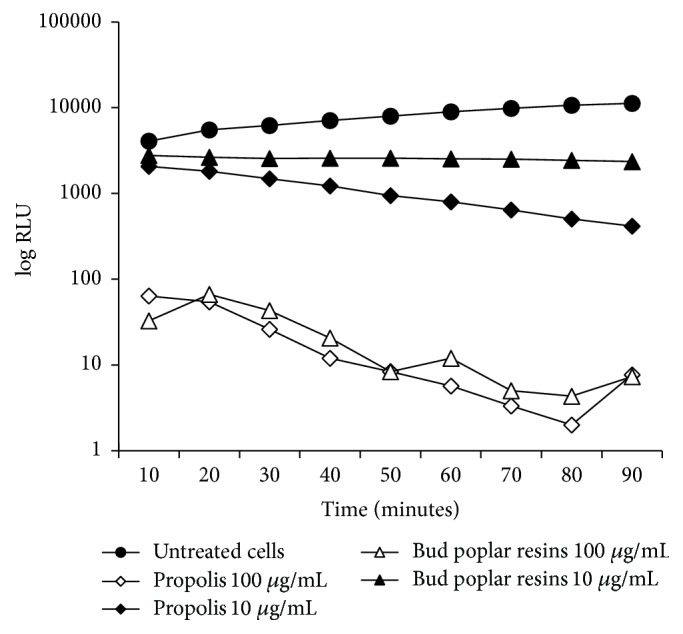
Antioxidant activity of different concentrations of propolis and bud poplar resins extracts on ROS production in human neutrophils. Antioxidant activity of different concentrations of extracts on ROS production in human neutrophils stimulated with PMA has been determined by luminol-dependent chemiluminescence assay. Results are expressed as Log of RLU (relative luminescence units). The figure is representative of two independent experiments with similar profiles performed in triplicate.

Total ROS production by human neutrophils stimulated with phorbol-12-myristate-13-acetate (PMA) has been tested by chemiluminescence assay. Neutrophils activated by PMA produce luminol-dependent chemiluminescence profiles following the ROS production after addition of the stimulus. The results obtained showed that propolis and bud poplar resins extracts were able to reduce ROS production by neutrophils activated with PMA in a dose-dependent manner (Figure 7).

### 3.6. Anti-Inflammatory Activity

**Figure 8:**
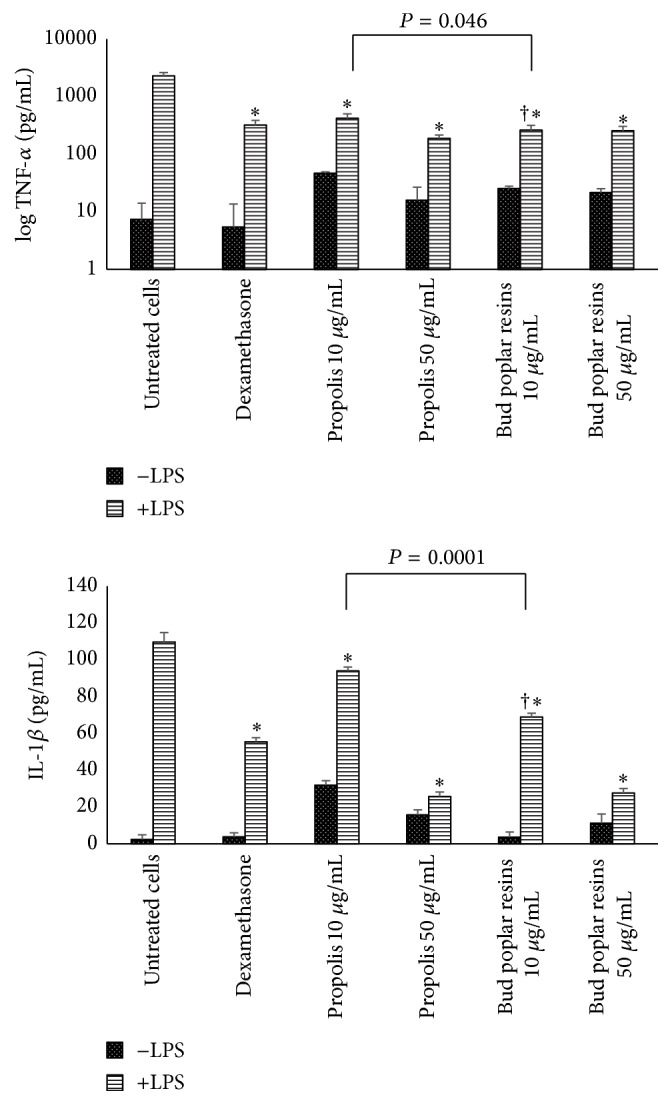
Anti-inflammatory activity of propolis and bud poplar resins extracts. TNF-*α* and IL-1*β* production by PBMC in response to LPS in presence of propolis or bud poplar resins extracts. PBMC were stimulated for 4 h with LPS (1 *μ*g/mL) and then treated overnight with extracts at the concentration of 10 and 50 *μ*g/mL. After incubation, supernatants were recovered and tested for the presence of TNF-*α* and IL-1*β* by ELISA assay. Data are expressed as mean ± SEM for three samples pooled from three independent experiments. ^*∗*^*P* < 0.05 (propolis plus LPS-treated cells versus LPS-treated cells) and ^†^*P* < 0.05 (bud poplar resins extract plus LPS-treated cells versus propolis extract plus LPS-treated cells). Differences were analyzed by *t* test.

Since propolis mechanisms of action are not completely clear, its downregulating effect on proinflammatory cytokine production has been described. TNF-*α* and IL-1*β*, secreted by immune cells, play an important role in directing the course of the inflammatory response. We analyzed if propolis and bud poplar resins extracts were able to reduce the proinflammatory cytokines secretion by human leukocytes stimulated by bacterial LPS. Propolis and bud poplar resins extracts were both able to inhibit the TNF-*α* and IL-1*β* secretion by human leukocytes stimulated with LPS at both concentrations tested (Figure 8). Moreover, bud poplar resins extract was able to reduce the production of both cytokines better than propolis extract at the concentration of 10 *μ*g/mL. Of note is the ability of both extracts to downregulate the production of proinflammatory cytokines in a similar manner to the positive control dexamethasone.

## 4. Discussion

The biological, physiological, and medicinal benefits of propolis have been extensively studied and reviewed in literature [26]. Recently, the antimicrobial activity of polish propolis against a* P. aeruginosa* strain has been reported [27]. Propolis antibacterial property has been attributed to phenolic compounds, especially flavonoids, phenolic acids, and their esters [28]. The antimicrobial activity of propolis is a result of a synergistic action between flavonoids and other compounds present in these extracts [29]. Moreover, propolis produced by* Melipona fasciculata* can exert antimicrobial action against* Streptococcus mutans* and* Candida albicans*, with significant inhibitory activity against* S. mutans* biofilms and displays anti-inflammatory effect [30]. Ethanolic extract of geopropolis collected by* Melipona scutellaris* did not inhibit the growth of* P. aeruginosa* [31].

Our data highlight that propolis and bud poplar resins extracts hold the same antibacterial activity against* P. aeruginosa* (MIC of 125 *μ*g/mL) even though the kinetics of the growth showed that bud poplar resins extract is able to delay the growth with respect to bee propolis.

Bacteria have two life forms: planktonic cells and bacteria organized into sessile aggregates (biofilm). Acute infections involving planktonic bacteria are generally treatable with antibiotics. However, when the bacteria succeed in forming a biofilm, the infection often turns out to be untreatable and will develop into a chronic state, in which bacteria become extremely resistant to antibiotics and conventional antimicrobial agents, and they acquire the ability to evade the host defenses [32]. Neovestitol-vestitol fraction contained in the Brazilian red propolis inhibits the biofilm development of* S. mutans* in in vitro and in in vivo models [33, 34]. Moreover, it has been demonstrated that propolis negatively interacts with* S. aureus* adhesion and biofilm formation by inhibiting virulence factors such as lipase and coagulase [35]. Therefore, the propolis and bud poplar resins extracts were tested on* P. aeruginosa* biofilm formation. Both extracts have shown the ability to reduce the biofilm formation in a dose-dependent manner at sub-MIC concentrations. The antibiofilm activity of both extracts was partially due to the inhibition of swimming activity of* P. aeruginosa*. Bud poplar resins extract was also able to increase swarming motility involved in the progression of biofilm maturation process. To our knowledge, we report for the first time the ability of both propolis and bud poplar resins extracts to reduce the biofilm formation of* P. aeruginosa*

Then we tested the cytotoxic effects of propolis and bud poplar resins extracts on a panel of different cell lines. The strongest cytotoxic activity has been observed for BEAS-2B cells (CC_50_ 73.6 and 58.00 *μ*g/mL for propolis and bud poplar resins extracts, resp.). The results obtained for the cell line A549 are very similar to that observed by Kouidhi et al. [36].

It is known that propolis is capable of dose dependently suppressing phytohemagglutinin- (PHA-) induced DNA synthesis of PBMC and proinflammatory cytokine production probably via immunoregulatory T cells [37]. Therefore, in addition to the antibacterial, antibiofilm, and cytotoxic activities, immunomodulatory effects of propolis and bud poplar resins extracts have been tested. We analyzed the anti-inflammatory activity of propolis and bud poplar resins extracts on human peripheral blood mononuclear cells stimulated with the typical Gram-negative constituent lipopolysaccharide. We observed a downregulation of IL-1*β* and TNF-*α* production. These data confirm the data obtained by Liberio et al. in a mouse model; they observed that geopropolis increased production of IL-4 and IL-10 and cytokines associated with a Th2 response, suggesting an anti-inflammatory activity [30]. The antioxidant components of extracts could influence the proinflammatory immune response, particularly due to their suppressive effect observed on PBMC. Then, the antioxidant property of propolis and bud poplar resins extracts has been tested by a cell free DPPH assay and by the quantification of ROS produced by human PMN. Both extracts showed a dose-dependent antioxidant activity.

Interestingly, the different percentages of flavonoids observed in propolis and bud poplar resins could account for their different in vitro effects. For example, the stronger activity of propolis extract in reducing TNF-*α* and IL-1*β* secretion and inhibiting ROS production could be due to its higher content in chrysin and galangin compared to bud poplar resins extract, while their comparable antimicrobial activity could reflect their similar content in pinocembrin. Indeed, while chrysin and galangin have been shown to hold effective antioxidant and anti-inflammatory activity [38–42] pinocembrin is considered to possess antimicrobial properties [43, 44]. The similarity in the composition and the activity between both extracts (from propolis and buds) is due to the fact that poplar trees are the source of the propolis sample. However, the observed differences suggest how the saliva of bees can influence the biological activities of propolis.

In conclusion, the potential use of propolis and bud poplar resins extracts as adjuvant in the therapy against* P. aeruginosa* chronic infection is promising not only for its antibiofilm activity, but also for its biological properties as anti-inflammatory and antioxidant properties and its low toxicity.

## Figures and Tables

**Table 1:** Phytochemical analysis of propolis and poplar bud resins extracts.

	Poplar buds freeze-dried extract (%)	Italian propolis freeze-dried extract (%)
Total flavonoids expressed as galangin	24.18 ± 0.80	28.78 ± 1.50
Chrysin	3.30 ± 0.20	3.50 ± 0.74
Galangin	2.74 ± 0.20	3.58 ± 0.60
Pinocembrin	3.16 ± 0.20	2.74 ± 0.40
Caffeic acid phenethyl ester (CAPE)	1.40 ± 0.03	1.52 ± 0.10

**Table 2:** Antibacterial activity of propolis, bud poplar resins extracts, and gentamicin on different *Pseudomonas aeruginosa *strains.

	*Pseudomonas aeruginosa P1242*		*Pseudomonas aeruginosa PAO-1*	
	MIC (*μ*g/mL)	MBC (*μ*g/mL)	MIC (*μ*g/mL)	MBC (*μ*g/mL)
Propolis	125.0	>2000	125.0	>2000
Buds poplar resins	125.0	>2000	125.0	>2000
Gentamicin	1.8	3.9	0.9	3.9

MIC (minimal inhibitory concentration) and MBC (minimal bactericidal concentration) were determined in three independent experiments.

**Table 3:** Cytotoxic concentration (CC_50_) of different extracts on HeLa, BEAS-2B, and A549 cell lines and human monocytes.

CC_50_ (*μ*g/mL)	Propolis	Bud poplar resins
*HeLa*	108.25	110.89
*BEAS-2B*	73.6	58.0
*A549*	175.8	163.8
*Human PBMC*	71.3	88.5

Cytotoxicity was tested by the determination of the cell ATP level by a bioluminescent method after 24 h of incubation. CC_50_ is the concentration required to reduce the live cell number by 50% compared to the untreated controls.
